# Removal of movement artifacts and assessment of mental stress analyzing electroencephalogram of non-driving passengers under whole-body vibration

**DOI:** 10.3389/fnins.2024.1328704

**Published:** 2024-04-25

**Authors:** Byoung-Gyu Song, Namcheol Kang

**Affiliations:** ^1^Department of Mechanical Engineering, Kyungpook National University, Daegu, Republic of Korea; ^2^School of Mechanical Engineering, Kyungpook National University, Daegu, Republic of Korea

**Keywords:** adaptive filter, electroencephalogram, mental stress, movement artifact, whole-body vibration

## Abstract

The discomfort caused by whole-body vibration (WBV) has long been assessed using subjective surveys or objective measurements of body acceleration. However, surveys have the disadvantage that some of participants often express their feelings in a capricious manner, and acceleration data cannot take into account individual preferences and experiences of their emotions. In this study, we investigated vibration-induced mental stress using the electroencephalogram (EEG) of 22 seated occupants excited by random vibrations. Between the acceleration and the EEG signal, which contains electrical noise due to the head shaking caused by random vibrations, we found that there was a strong correlation, which acts as an artifact in the EEG, and therefore we removed it using an adaptive filter. After removing the artifact, we analyzed the characteristics of the brainwaves using topographic maps and observed that the activities detected in the frontal electrodes showed significant differences between the static and vibration conditions. Further, frontal alpha asymmetry (FAA) and relative band power indices in the frontal electrodes were analyzed statistically to assess mental stress under WBV. As the vibration level increased, EEG analysis in the frontal electrodes showed a decrease in FAA and alpha power but an increase in gamma power. These results are in good agreement with the literature in the sense that FAA and alpha band power decreases with increasing stress, thus demonstrating that WBV causes mental stress and that the stress increases with the vibration level. EEG assessment of stress during WBV is expected to be used in the evaluation of ride comfort alongside existing self-report and acceleration methods.

## 1 Introduction

A large number of occupants are exposed to vertical vibrations in various forms of transport such as automobiles, aircraft, trains, and ships. These vibrations, known as whole-body vibrations (WBVs), are transmitted to either specific parts or the entire human body through the seat structure, which consists of the seat surface, backrest, and foot area. The WBV during driving can cause adverse effects in occupants, including motion sickness (Donohew and Griffin, 2004), drowsiness (Bhuiyan et al., 2022), low-back pain (Burström et al., 2015), and discomfort (Griffin, 2007). Of these effects, discomfort is particularly noteworthy as it is becoming more important as long-distance travel increases with advances in autonomous driving and vehicle infotainment technologies. Therefore, there is an emerging need to quantitatively measure and assess discomfort in WBV environments from a brain response perspective.

The discomfort caused by vertical WBV has long been assessed using a self-report questionnaire or acceleration. In the self-report questionnaire, discomfort has been quantified by subjective ratings (often on a scale of 1–10) from test drivers during product development (Griffin, 2007; Badiru and Cwycyshyn, 2013; Taluja et al., 2017). In addition, several researchers have used the equivalent comfort contour to quantify discomfort in terms of the magnitude, frequency, and direction of the vibration (Griffin and Erdreich, 1991; Morioka and Griffin, 2006; Zhou and Griffin, 2014; Huang and Zhang, 2019; Lin et al., 2022). The equivalent comfort contour is a representation of the physical magnitude of a WBV that causes the same level of discomfort over a range of frequencies. It is obtained by subjective ratings of the magnitude and frequency of the vibration (Zhou and Griffin, 2014). The equivalent comfort contour has been used as a guideline for human factor design in transport, as it indicates which frequency ranges are sensitive and how this varies with magnitude. While these methods are useful for making relative judgments about the ride comfort of different vehicles, they are inadequate for providing an absolute measure of comfort (Griffin, 2007). Subjective ratings are not as sensitive to small changes in vibration; for example, reductions in vibration magnitude of less than 10% are generally undetectable by subjective ratings (Morioka and Griffin, 2000). Meanwhile, in the acceleration-based method, the international standards ISO 2631-1 and BS-6841 proposed a ride index to quantify discomfort by applying a frequency weighting and axis multiplication factors to accelerations measured at different locations on the body (Griffin and Erdreich, 1991; Griffin, 2007; Badiru and Cwycyshyn, 2013; Taluja et al., 2017). Several studies have used the ride index to evaluate discomfort in different modes of transport, such as a car (Cantisani and Loprencipe, 2010; Song et al., 2023), a train (Zoccali et al., 2018), a helicopter (Delcor et al., 2022), and a cruise flight (Huang and Li, 2023). However, the acceleration-based rating has the disadvantage that it cannot reflect within-subject variability, such as personal preferences that vary across different environments, and between-subject variability, such as exceptional sensitivity in certain passengers. Additionally, the human response, which is influenced by stress factors, cannot be accurately measured by acceleration alone.

Physiological signals have been considered potential objective tools to detect emotional changes and stress of individuals (Alarcao and Fonseca, 2017; Giannakakis et al., 2022). In particular, electroencephalogram (EEG) signals are advantageous over other physiological signals in detecting individual emotions because cognitive status is directly associated with brain activity (Cheng et al., 2022). Numerous studies have used EEG to detect individual emotional changes in response to different driving conditions. For instance, Rebolledo-Mendez et al. (2014) proposed a detector of human emotions such as tiredness and stress (tension), which are highly related to traffic accidents in highway and urban environments. They used a body sensor network (BSN) consisting of EEG and electrodermal activity sensors to detect emotions. They showed that it is possible to detect one emotional state in real time using BSN. Halim and Rehan (2020) presented a machine learning-based approach based on EEG to identify stress patterns caused by driving. To predict EEG patterns based on subjects’ self-reported emotional state during various driving situations, they used Support Vector Machine, Neural Network, and Random Forest. This study demonstrated that the spectral power of differential hemispheric asymmetry set for five frequency bands, including alpha, low beta, high beta, gamma, and theta, is an appropriate metric for distinguishing brain dynamics in response to stressful stimuli. Hussain et al. (2021) investigated mental workload-induced neurological changes in 17 healthy male drivers using a portable EEG headset while driving in different driving scenarios. The extent of change varied depending on the type of road, speed and signal regulations, the behavior of surrounding vehicles, and the overall traffic situation. This study also interpreted EEG features in driving workload. Affanni et al. (2022) presented a six-channel EEG wearable headband to measure discomfort related brain activity during driving. The spectral power of the beta wave was used as an indicator of discomfort. Prior research has predominantly concentrated on the mental stress experienced by drivers, with less attention given to that of passengers. Passengers, however, may face considerable stress owing to vibrations from road surface roughness, while a driver’s stress stems from the demands of the driving task itself. To our knowledge, there has yet to be a published study investigating the mental stress of passengers caused by whole-body vibration (WBV), as analyzed through EEG.

In EEG measurement, WBV can cause movement artifacts. Due to difficulty of removing these artifacts, many studies may not have considered whole-body vibration in driving situations. However, in the field of gait analysis, several methods have been used to remove these artifacts. For instance, Gwin et al. (2010) used independent component analysis (ICA) and component-based template regression to remove gait movement artifacts. Some studies have used artifact subspace reconstruction (ASR) and ICA to remove artifacts that occur during walking (Bulea et al., 2015; Oh et al., 2021). ICA-based methods typically necessitate human intervention to identify and remove artifact components. Consequently, expert knowledge is requisite to discern movement artifacts for elimination, posing a challenge for real-time implementation. In contrast, adaptive filters can remove movement artifacts autonomously without human involvement, enabling automation as long as the acceleration signals corresponding to the movement artifacts are measured. Several studies have successfully employed adaptive filters to remove the fundamental frequency of contamination, synchronized with walking cadence, and its associated harmonics from EEG signals collected during ambulation (Mihajlović et al., 2014; Kilicarslan and Vidal, 2019; Rosanne et al., 2021). However, no studies to date have addressed the removal of movement artifacts from EEG recordings of seated individuals exposed to vertical whole-body vibration.

In this study, we verified the performance of an adaptive filter for WBV-induced movement artifacts in the EEG signal when subjected to the vertical random vibration. Herein, we used the reference channel of the adaptive filter as the vertical acceleration signal measured at the head. Moreover, to determine the optimal adaptive filter method, we compared the performance of a normalized least mean square (NLMS) adaptive filter and a recursive least square (RLS) adaptive filter for artificial movement artifacts. After artifact removal using an adaptive filter, the spatial characteristics of the EEG signal were analyzed using topographic maps. We also analyzed the changes in the EEG signal of occupants with respect to the magnitude of the random vibration in terms of stress. For stress analysis, we used the frontal alpha asymmetry (FAA) and relative power indices. These indicators were compared and analyzed with results from the existing literature on stress analysis.

## 2 Materials and methods

### 2.1 Participants

A total of 25 volunteers (13 males and 12 females, age: 22.64 ± 1.93) participated in this study. However, data from three participants (2 males and 1 female) were excluded due to corrupted EEG recordings, leaving 22 participants (11 males and 11 females, aged 22.68 ± 2.06 years) whose data were analyzed. These participants were selected as individuals without any physical or psychological issues. They were required to abstain from alcohol and to get sufficient sleep the day before the experiment, which could otherwise the experimental results. They were also instructed not to consume caffeine 3 h before the start of the experiment and provided a detailed explanation of the objectives and procedures of the experiment, in advance, after which all of them signed an informed consent. Monetary compensation was provided for their participation after the experiment. The overall experimental protocol was reviewed and approved by the institutional review board at Kyungpook National University (2023-0041), 27 January 2023.

### 2.2 Experimental environment

**Figure 1:**
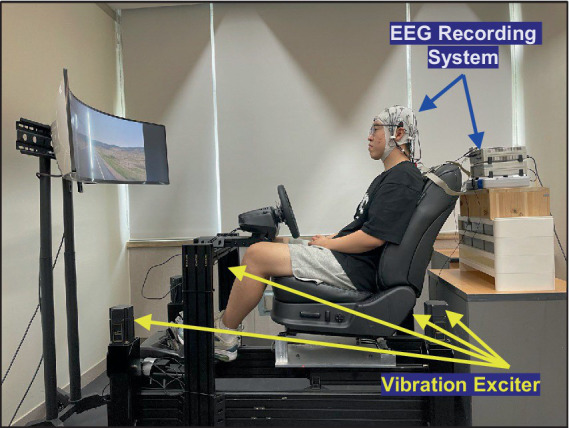
Experimental environment to collect EEG data with respect to vertical whole-body vibration.

To explore the neurophysiological responses to random vibrations, the participants were exposed to the vibrations using a motion simulator, as depicted in Figure 1. The random vibrations were characterized by an approximately flat constant bandwidth spectrum, constrained by a Butterworth filter with cutoff frequencies set at 0.5 and 25 Hz. The magnitude of the vibration was established at 1.0, 1.5, and 2.0 m/s^2^ based on the root mean square (r.m.s.). To mimic conditions similar to real-world vehicular operation, the participants watched a road-view video during the excitation of vibration.

To reduce non-vibratory stressors, several precautions were explained before the experiment. Participants were allowed a rest period to ensure they were well rested. The laboratory was maintained at a constant room temperature to provide a comfortable environment. In addition, to minimise the effects of ambient noise and discomfort due to sitting posture, participants were provided with earplugs and instructed to maintain an upright posture, ensuring that their backs were fully supported by the backrest.

**Figure 2:**
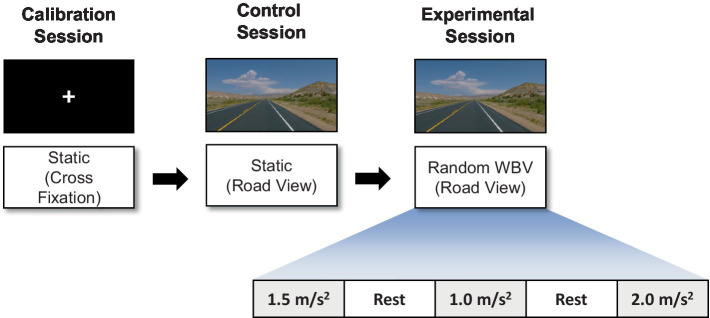
Illustration of the experimental paradigm: calibration, control, and experimental sessions.

The vibration test was organized into three sessions: a calibration session, a control session, and an experimental session as shown in Figure 2. During the calibration session, which was designed for the collection of EEG data for artifact removal, the participant watched a white fixation cross with a black background for 2 min. In the control session, which was designed to collect data as a baseline for subsequent comparisons, the participant watched a road-view video without any vibration for 2 min. In the experimental session, the participants watched the road-view video under a vertical random vibration for 2 min and 10 s. The order of the vibrations was randomized to exclude any preconceptions about the magnitude of the excitation. Finally, a 1 min rest interval was incorporated between each test to minimize the influence of the previous experiment on the next.

### 2.3 Data acquisition

**Figure 3:**
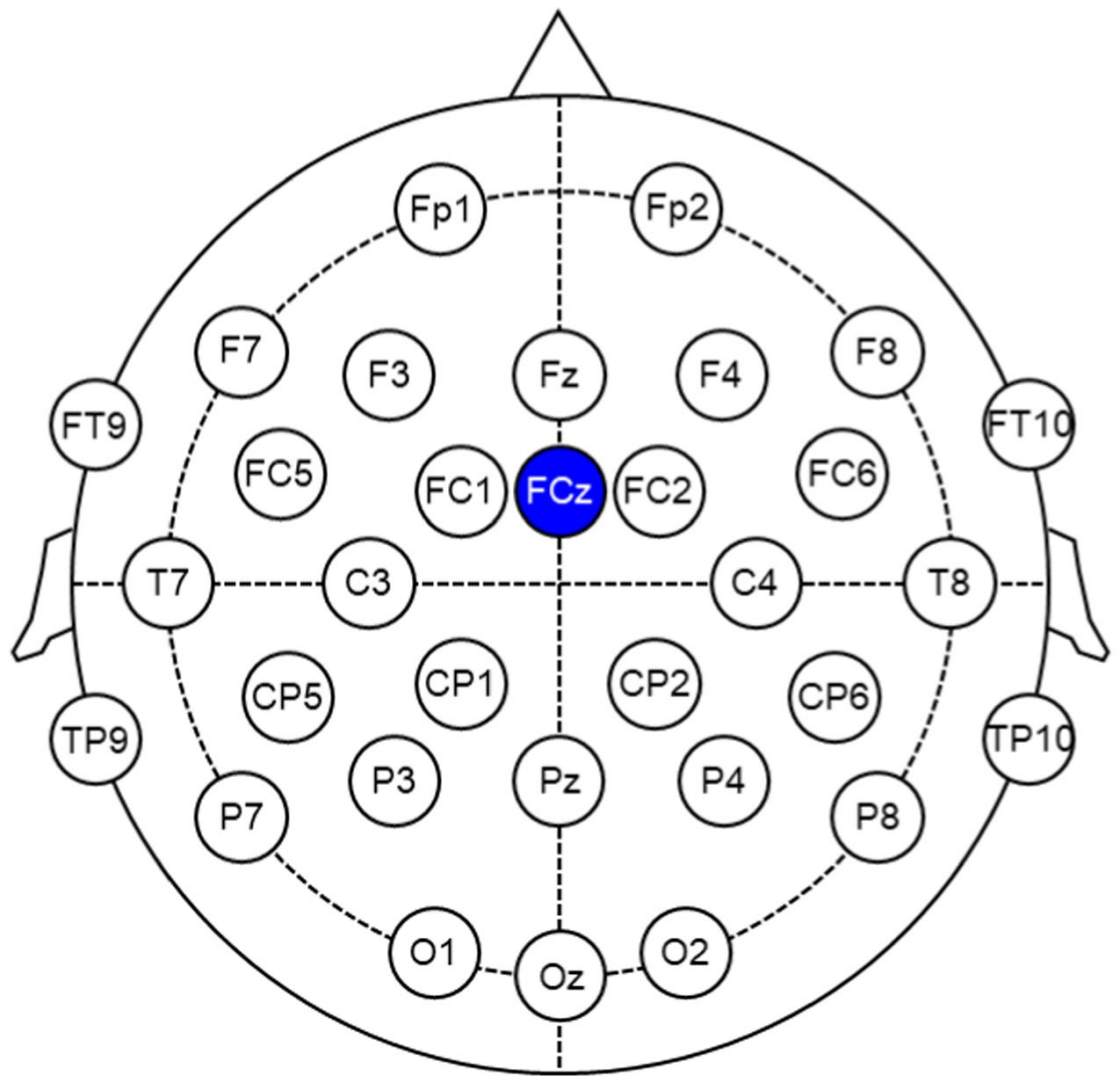
EEG channel layout based on the 10–20 international system. The reference channel is FCz.

EEG signals were recorded using actiCHamp Plus (Brain Vision, Morrisville, NC, United States) at a sampling rate of 500 Hz. The 32 channel EEG electrodes were placed on the participant’s scalp according to the 10–20 international system to record EEG signals (Fp1, Fp2, F7, F3, Fz, F4, F8, FT9, FC5, FC1, FCz, FC2, FC6, FT10, T7, C3, C4, T8, TP9, CP5, CP1, CP2, CP6, TP10, P7, P3, Pz, P4, P8, O1, Oz, and O2) as depicted in Figure 3. The reference channel was FCz, and the impedance of all electrodes was maintained below 10 kΩ . Additionally, we measured the head acceleration to eliminate the movement artifact caused by WBV.

### 2.4 Preprocessing

**Figure 4:**
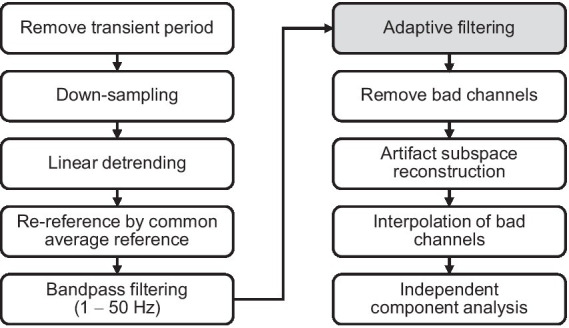
EEG preprocessing pipeline for artifact removal.

Data preprocessing was performed using MATLAB (MathWorks, Natick, MA, United States) and the EEGLAB toolbox (Delorme and Makeig, 2004) as illustrated in Figure 4. An initial segment of EEG data from 0 to 10 s was eliminated not to consider the transient response induced by the vibration. The EEG signals were down-sampled from 500 to 250 Hz. The linear trends of EEG signals were subtracted from each EEG signal to remove signal drift. The EEG signals were then re-referenced to the common average of all channels to remove common noise and bandpass-filtered from 1 Hz to 50 Hz using a finite impulse response filter. To remove movement artifacts in the EEG signals, we used an adaptive filter. The adaptive filter was demonstrated in the following subsection E. Bad channels were detected and removed based on standard deviation and correlation between channels. Next, we used the artifact subspace reconstruction (ASR) algorithm (Mullen et al., 2013; Pion-Tonachini et al., 2018) to detect high-variance data epochs in each channel, and after removing them, we reconstructed the missing data by referring to the reference data. In this study, 20 standard deviation was used as a variance threshold. Herein, the reference data is the EEG data measured in the calibration session. Thereafter, the previously removed channel was interpolated using the data of adjacent channels. Finally, we performed an extended ICA (Jung et al., 1997; Zhou and Gotman, 2004) for the extraction and removal of extraneous artifacts (eye blink, eye movement, muscle activity, and heartbeats) from the EEG data. The type of independent component (brain, eye, muscle, heartbeat, etc.) was determined automatically using EEGLAB’s ICLabel (Pion-Tonachini et al., 2019). A specific type was removed when the probability of that type was greater than 80%, except for the brain.

### 2.5 Movement artifact removal using adaptive filters

**Figure 5:**
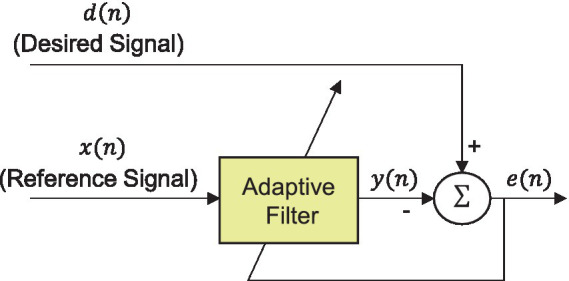
Block diagram of an adaptive filter to remove movement artifacts.

Movement artifacts caused by body and head movements during signal measurement are a major obstacle in EEG analysis. These artifacts are predominantly distributed within the frequency range below 10 Hz, overlapping with the EEG frequency band (Mihajlović et al., 2014; Kilicarslan and Vidal, 2019; Rosanne et al., 2021). Therefore, to remove the artifacts induced by WBV, we adopted an adaptive filter, which is a linear filter whose transfer function is controlled by variable parameters and a system that can adjust these parameters according to an optimization algorithm. The architecture of the adaptive filter utilized in this study was designed in the block diagram depicted in Figure 5. The adaptive filter can be represented as follows Equation (1):


(1) $$y\left[n\right]=\overset{L}{\underset{k=0}{\sum }}w_{k}x\left[n-k\right]$$

where *y* [*n*] indicates the movement artifact estimated by the filter, *L* denotes the order of the filter, and *x* [*n*] is the reference signal, which corresponds to the head acceleration measured during WBV. Also, *w_k_* represents the filter coefficient. The error for optimizing the filter is defined as the difference between the noisy EEG signal, *d* [*n*], and the estimated movement artifact, *y* [*n*] can be expressed as follows Equation (2):


(2) $$e\left[n\right]=d\left[n\right]-y\left[n\right]$$

In this study, we used NLMS and RLS algorithms to identify the optimal weights of the filter that minimizes the error (Haykin, 2014). The noise cancellation performance of the two methods was compared under various noise conditions, as described in the Results section.

## 3 Results

### 3.1 Application of adaptive filters to artificial noise signal

To evaluate the performance of the adaptive filters, we generated the artificial noise signal which is expressed as a linear combination of a pure EEG and measured head acceleration signals. The mathematical representation of the noisy EEG signal is given by Equation (3)


(3) $$\hat{d}\left[n\right]=s\left[n\right]+\lambda N\left[n\right]$$

where $\hat{d}$[*n*] denotes the EEG signal artificially contaminated by the movement artifact, and *s*[*n*] and *N*[*n*] denote the pure EEG signal as ground truth and movement artifact, respectively. The movement artifact used in this study is the vertical head acceleration with a magnitude of 2.0 m/s^2^ r.m.s. Also, λ represents the hyperparameter to modulate the signal-to-noise ratio (SNR) in the contaminated signal. The SNR can be mathematically expressed as follows Equation (4):


(4) $$SNR=10\;\log\frac{RMS\left(s\right)}{RMS\left(\lambda \;N\right)}$$

where RMS indicates the root mean square of a signal. The smaller the SNR, the higher the noise level. In this study, we evaluated the performance of the adaptive filter for the noisy EEG signals of two different SNRs: −8 dB and 4 dB.

We then qualitatively evaluated the performance of the adaptive filters using the relative root mean squared error (RRMSE) in the temporal and spectral domains, the correlation coefficient (CC), and the relative band power as follows Equation (5), (6) (Zhang et al., 2021):


(5) $${\mathrm{RRMSE}}_{\mathrm{temporal}}=\frac{RMS\left(f\left(\hat{d}\right)-s\right)}{RMS\left(s\right)}$$

(6) $${\mathrm{RRMSE}}_{\mathrm{spectral}}=\frac{RMS\left(PSD\left(f\left(\hat{d}\right)\right)-PSD\left(s\right)\right)}{RMS\left(PSD\left(s\right)\right)}$$

where the function *f* (•) and PSD (•) denote the filtered signal by the adaptive filter and the power spectral density of an input signal. PSD was estimated using Welch’s method (Welch, 1967). The window length was set to 2 s, and the overlap was set to 50%. The frequency range of PSD is 1–50 Hz. The CC is Equation (7)


(7) $$CC=\frac{\mathrm{cov}\left(f\left(\hat{d}\right),s\right)}{\sqrt{Var\left(f\left(\hat{d}\right)\right)Var\left(s\right)\;}}$$

A correlation close to unity implies that the filtered signal and the ground truth are exactly coincident. The relative band power (RP) is expressed as the power of the specific band divided by the total power of all bands and calculated as follows Equation (8):


(8) $$RP=\frac{\mathrm{power}\left(\mathrm{selected}\;\mathrm{band}\right)}{\mathrm{power}\left(\mathrm{total}\;\mathrm{bands}\right)}$$

The RP can minimize the bias that is caused by differences in the conductivity of the skull and scalp between individuals (Bronzino, 2000).

**Figure 6:**
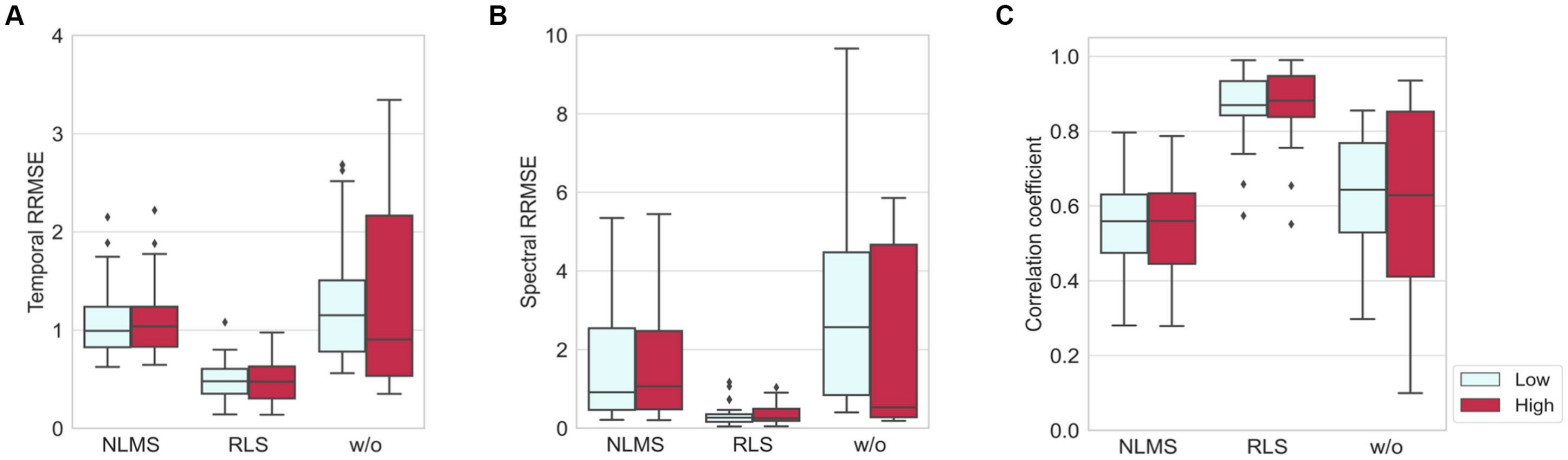
Comparisons of three methods, NLMS, RLS, and without adaptive filter, in the presence of artificial noise where low and high indicate when SNR = 4 dB and SNR = −8 dB, respectively: **(A)** temporal RRMSE, **(B)** spectral RRMSE, and **(C)** correlation coefficient.

The results of NLMS and RLS adaptive filters were compared with those without adaptive filters in terms of quantitative metrics (RRMSE_temporal_, RRMSE_spectral_, and CC) as shown in Figure 6. All these three cases followed the same preprocessing procedure shown in Figure 4, except for the adaptive filter. The window length was set to 1 s. The results showed that the adaptive filters, regardless of the noise level, presented lower temporal and spectral RRMSE values and smaller deviations than the values obtained when adaptive filters were not employed. On the other hand, in the case of CC, the results of the NLMS adaptive filter were lower than when no adaptive filter was used. However, the results of the RLS adaptive filter showed the highest value with the smallest deviation for CC. These results indicated that the RLS adaptive filter exhibits the greatest robustness and superior performance across the three benchmarks.

**Figure 7:**
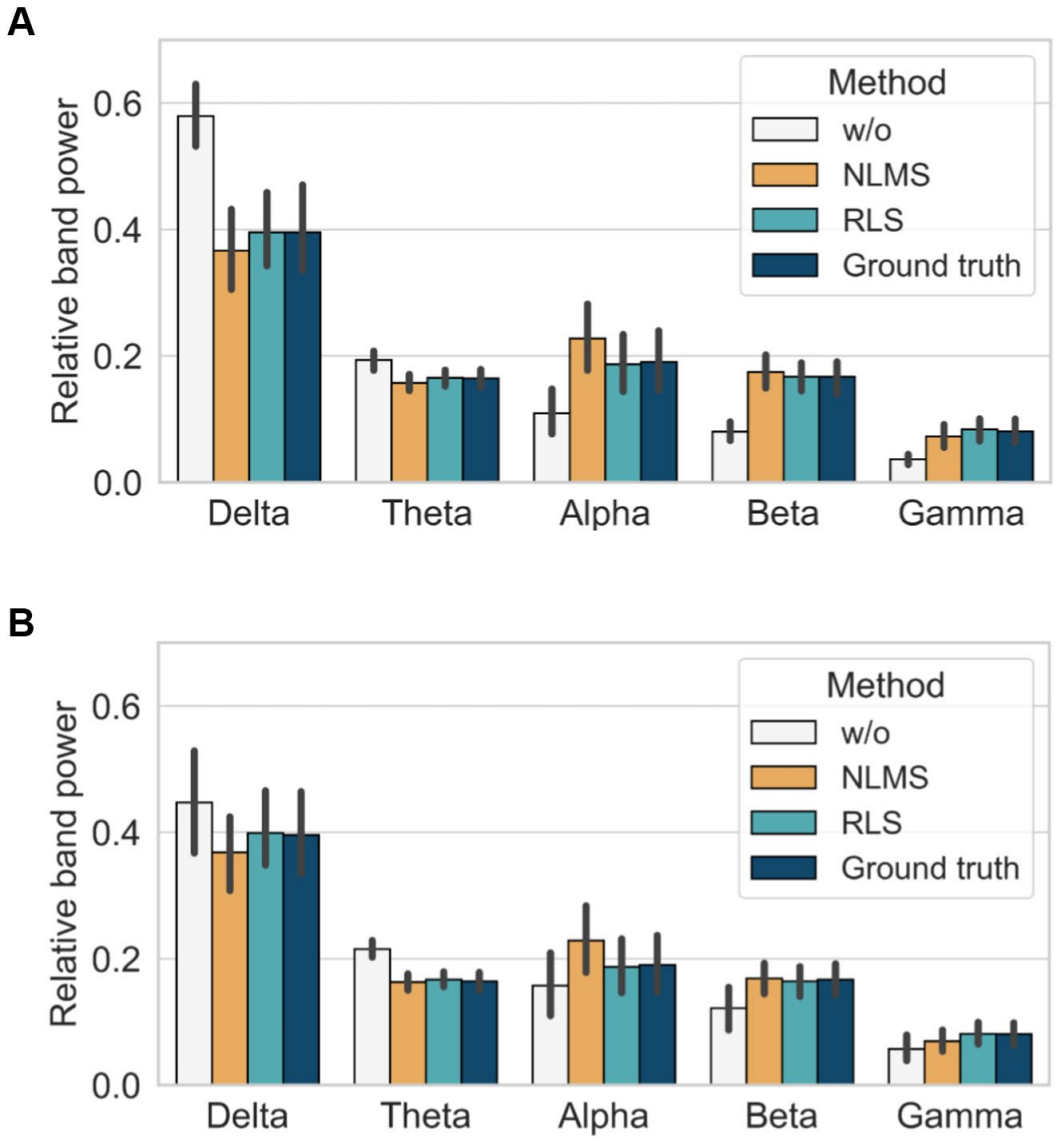
Comparison of relative band power with and without adaptive filters: **(A)** when SNR = 4 dB and **(B)** when SNR = −8 dB.

We finally assessed the performance of the different methods by calculating the RP over different frequency bands as follows: delta (1–4 Hz), theta (4–8 Hz), alpha (8–13 Hz), beta (13–30 Hz), and gamma (30–50 Hz) bands. In the case without the adaptive filter, a significant difference was observed in all bands compared with the ground truth, irrespective of the noise level, as demonstrated in Figure 7. In contrast, the RLS adaptive filter showed the closest results to those of the ground truth in all bands, irrespective of the noise level. Therefore, the comparison of the performance of the adaptive filters using the three quantitative metrics and relative power indices revealed that the RLS adaptive filter is superior in terms of accuracy and robustness.

### 3.2 Application of adaptive filters to real EEG signal

**Figure 8:**
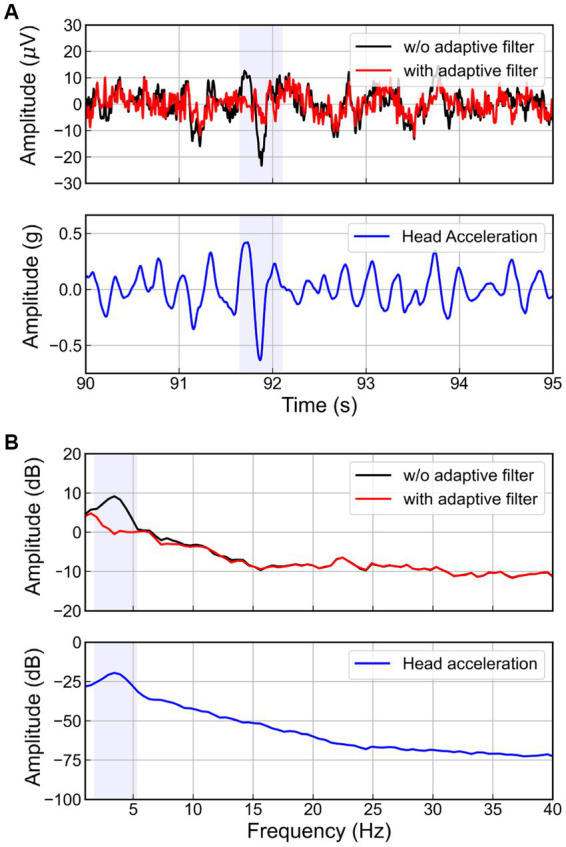
Head acceleration and EEG signals at FC1 channel in **(A)** time and **(B)** spectral domain with and without adaptive filters.

The RLS adaptive filter was applied to the actual EEG signal contaminated by the WBV. Figure 8 presents the results of applying the adaptive filter to the EEG on the FC1 channel of one subject when the magnitude of the random vibration is 2.0 m/s^2^ r.m.s. As highlighted blue box in Figure 8A, when the vertical head acceleration fluctuated severely due to head movement, we also observed a spike in the EEG for the case of without adaptive filter. On the other hand, the EEG signal filtered by the adaptive filter showed no spike. Considering this result in terms of the frequency domain (see Figure 8B), we observed a peak around 3.4 Hz in the head acceleration and EEG signal without the adaptive filter. Interestingly, this peak is similar to the natural frequency of the human body (Fairley and Griffin, 1989; Cho and Yoon, 2001; Zhou and Griffin, 2014). Meanwhile, the peak did not appear in the EEG signal filtered by the adaptive filter. Therefore, we confirmed that the adaptive filter successfully removed the movement artifacts corresponding to the resonance phenomenon of the human body.

In addition, to examine the correlation between head movement signals and EEG, we compared the performance of conventional artifact removal methods on all 22 participants with and without adaptive filters in terms of coherence. Coherence is defined as follows Equation (9) (Bendat and Piersol, 2011):


(9) $$\gamma_{xy}^{2}=\frac{{\left|S_{xy}\left(f\right)\right|}^{2}}{S_{xx}\left(f\right)\;S_{yy}\left(f\right)}$$

where $S_{xy}\left(f\right)$ is the cross-spectral density of the signals. Also, $S_{xx}\left(f\right)$ and $S_{yy}\left(f\right)$ are the PSDs of *x*(*t*) and *y*(*t*), respectively. A coherence of 0 indicates that no correlation exists between the two signals, whereas a coherence of 1 indicates that the two signals are fully correlated. In this study, we calculated the coherence between the vertical head acceleration and preprocessed EEG signals. Therefore, a higher coherence between the two reflects a significant inclusion of movement artifacts in the EEG signal.

**Figure 9:**
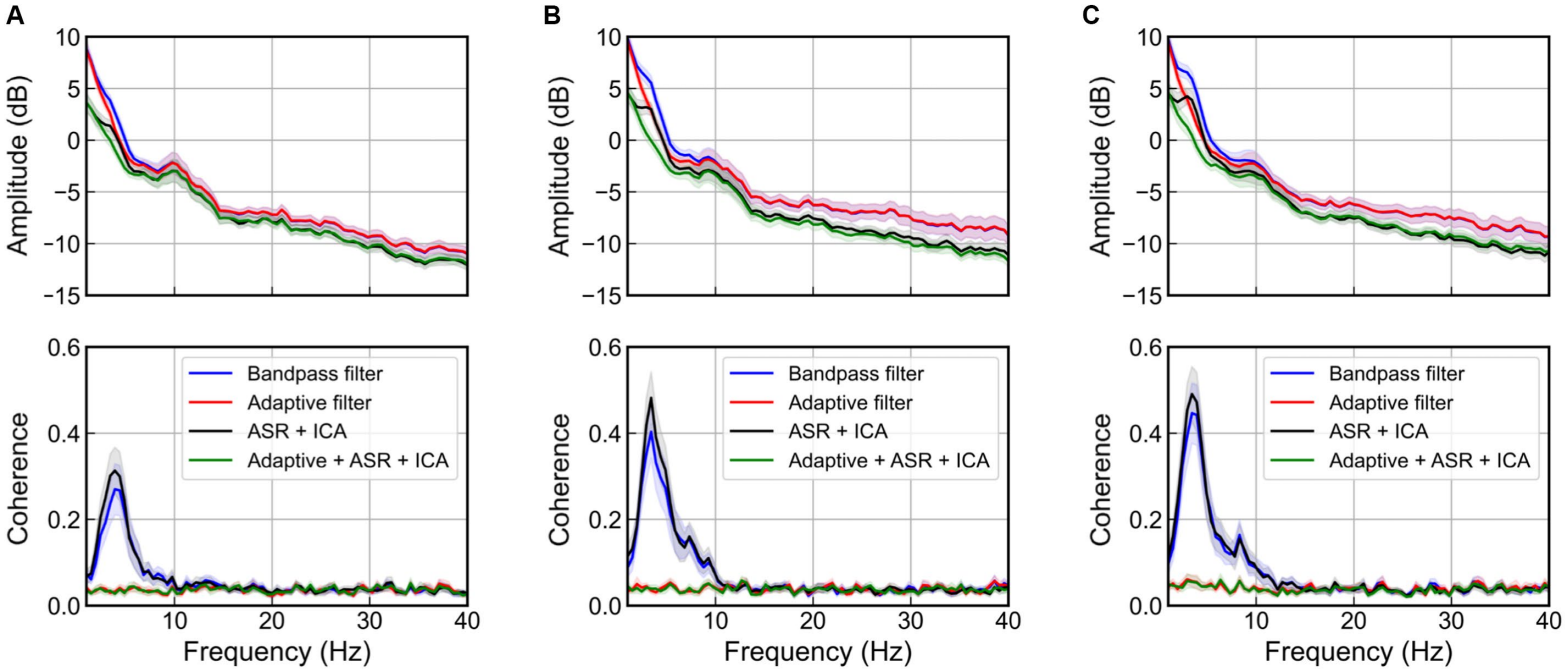
Power spectral density and coherence at the C3 channel when various artifact rejection methods are applied for the case of magnitude of random excitation are, respectively, **(A)** 1 m/s^2^ r.m.s., **(B)** 1.5 m/s^2^ r.m.s., and **(C)** 2 m/s^2^ r.m.s. The bold line indicates mean values, and the shaded area represents 95% confidence intervals of 22 participants.

Figure 9 shows the mean and 95% confidence interval of PSD and coherence at the C3 channel of the EEG, according to the magnitude of the vibration. In the absence of adaptive filter applications (bandpass filter and ICA after ASR), an increase in the magnitude of the excitation results in a larger peak near 4 Hz in the PSD as well as an increase in value in the 3–5 Hz range in coherence, due to movement artifacts. Although ASR and ICA can eliminate EOG artifacts below 4 Hz (Fatourechi et al., 2007) and EMG artifacts above 20 Hz (Muthukumaraswamy, 2013), they failed to remove the movement artifacts caused by WBV. Meanwhile, the adaptive filter successfully removed the movement artifact that occurred near 4 Hz and showed near-zero coherence, regardless of the magnitude of the excitation.

**Figure 10:**
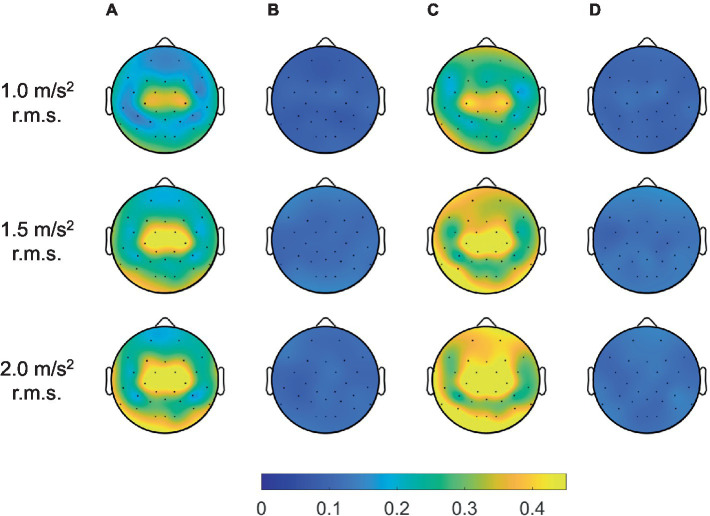
Grand mean coherence across all participants between head acceleration and EEG signals filtered by artifact rejection methods for all channels: **(A)** bandpass filter, **(B)** adaptive filter, **(C)** ICA with ASR, and **(D)** ICA with ASR after adaptive filter.

Furthermore, we considered the maximum coherence in the frequency range of all channels to verify the performance of the adaptive filter in terms of the spatial domain, as depicted in Figure 10. In the topographic maps, the color indicated average coherence for all participants. Herein, the blue and yellow colors indicated zero and high coherence, respectively. The methods without an adaptive filter showed high coherence near the center of the head. As the magnitude of the vibration increased, the region of high coherence expanded with increasing the coherence. Meanwhile, the methods with adaptive filters showed significantly near-zero coherence for all spatial domains regardless of vibration magnitude. Therefore, we confirmed that the adaptive filter successfully removed the movement artifacts caused by random WBV in temporal, spectral, and spatial aspects.

### 3.3 Mental stress with variations in vibration levels

Most studies on discomfort caused by WBV demonstrated that the level of discomfort increases with increasing magnitude of the vibration (Griffin, 2007). To confirm this, we investigated the frequency characteristics of the EEG signal when the magnitude of the vibration varied. Herein, we used only the final 30 s of the EEG signals measured during the 2 min for the stress assessment. Moreover, we considered the FAA, which is a reliable estimator of stress (Gatzke-Kopp et al., 2014). FAA is the difference in alpha’s power natural logarithm between the left and right frontal regions of the brain (Giannakakis et al., 2015), which is defined as follows Equation (10):


(10) $$\mathrm{Alpha}\;\mathrm{asymmetry}=\ln\left(\alpha \right)|{\;}_{F3}-\ln\left(\alpha \right)|{\;}_{F4}$$

Channels F3 and F4 are the regions directly affected by stress conditions and are commonly used to calculate alpha asymmetry (Qin et al., 2009).

**Figure 11:**
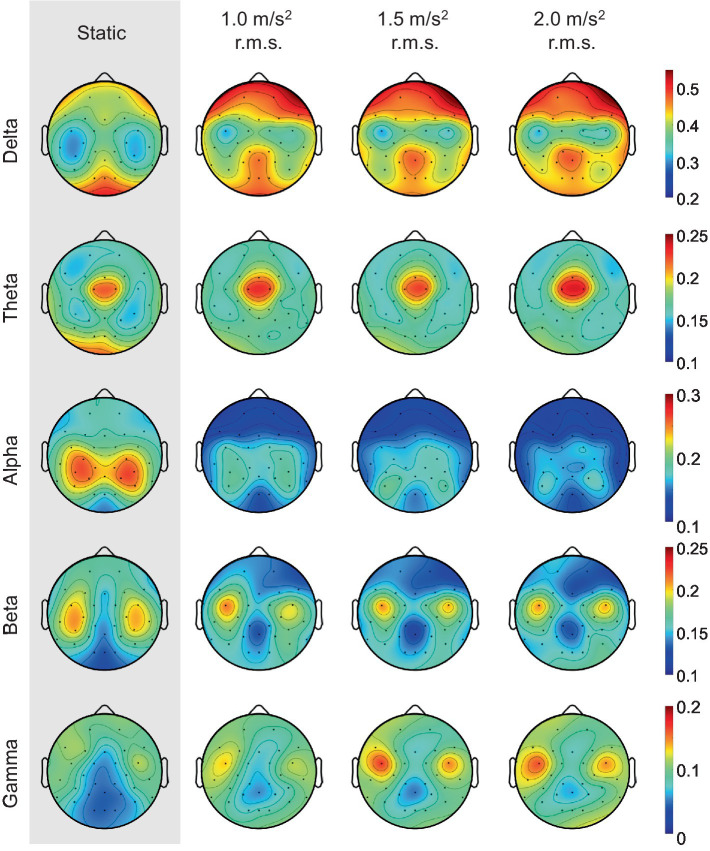
Grand mean topographic distribution of relative band powers across all participants as the magnitude of the vibration increases.

**Figure 12:**
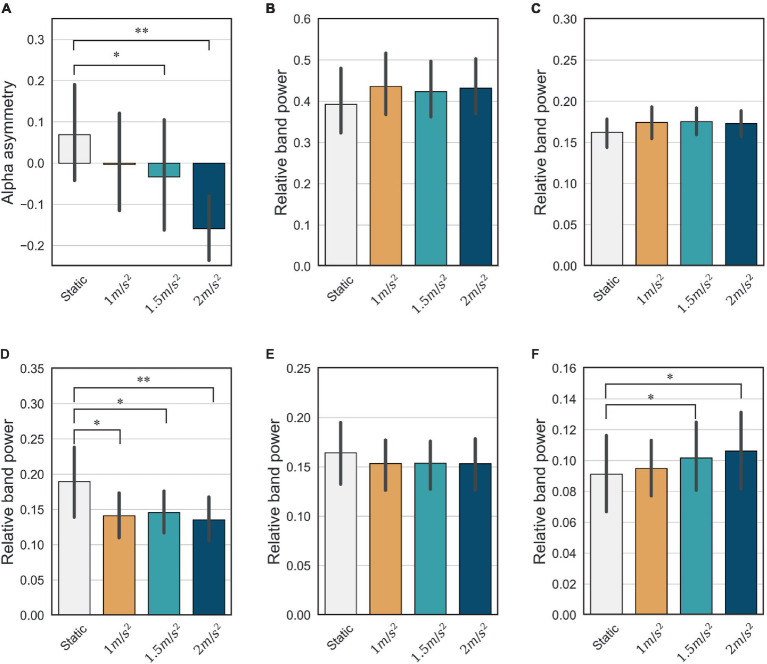
Mean and confidence intervals of alpha asymmetry and relative band power for all participants in the frontal lobe: **(A)** frontal alpha asymmetry **(B)** delta **(C)** theta **(D)** alpha **(E)** beta **(F)** gamma (* indicates *p* < 0.05 and ** indicates *p* < 0.01).

It is well known that the frontal lobe of the brain is associated with emotional regulation (Chayer and Freedman, 2001). As shown in Figure 11, this study also showed that the activities detected in the frontal electrodes exhibits the largest difference between the static and vibration conditions in each frequency band. Therefore, we investigated the changes in alpha asymmetry and RP in the frontal electrodes (Fp1, Fp2, F7, F3, Fz, F4, F8) between the no vibration (static) and vibration conditions, as shown in Figure 12. To analyze statistically significant differences between the static and vibration conditions, the Friedman test was performed first, followed by pairwise comparisons using the Durbin-Conover test. Jamovi software (version 2.3.18) was used for this purpose (The Jamovi Project, 2023). For FAA, as the magnitude of the excitation increased, the alpha asymmetry decreased, going negative. The static and 1.5 m/s^2^ r.m.s. conditions (*p* < 0.05) and the static and 2.0 m/s^2^ r.m.s. conditions (*p* < 0.01) were statistically different, respectively. For delta and theta bands, there was an increasing trend with increasing magnitude of the vibration, but this result was not statistically significant. For alpha band, the relative alpha band power decreased as the magnitude of the excitation increased. The static condition was statistically different from the 1.0 m/s^2^ r.m.s. condition (*p* < 0.05), and the difference between the static and vibration conditions significantly increased with increases in magnitude, e.g., when the magnitude is 2.0 m/s^2^ r.m.s., the *p*-value was less than 0.01. For beta band, there was a decreasing trend with increasing magnitude of the vibration, but this trend was not statistically significant. For gamma band, there was a trend of statistically increasing relative gamma band power with increases in magnitude (*p* < 0.05).

## 4 Discussion

### 4.1 Performance of adaptive filters on movement artifact

Vertical random vibration can contaminate the EEG signal by generating movement artifacts. We employed an adaptive filter to remove the movement artifacts. In order to find a suitable adaptive filter for our problem, we compared the performance of the normalized least mean square (NLMS) and recursive least square (RLS) adaptive filters in removing artificial movement artifacts in view of the relative root mean squared error (RRMSE) in the temporal and spectral domain, as well as the correlation coefficient (CC), and the relative power (RP). As shown in Figure 6, the RLS adaptive filter was the best and most robust across all matrices for both low and high noise levels. Without adaptive filters, the delta (1–4 Hz) and theta (4–8 Hz) band powers were higher than those of the ground truth, as shown in Figure 7. These two bands could be amplified due to the movement artifact caused by the vibrational resonance of the human body (Fairley and Griffin, 1989; Bronzino, 2000; Zhou and Griffin, 2014) when a seated person is subjected to random vibrations (see Figure 8). This implies that the relative powers of the other bands are significantly lower than the ground truth, as the sum of the relative powers does not change. The NLMS adaptive filter showed a lower value than that of the ground truth in the delta band, which can be considered to have removed both brain activity and movement artifacts. On the other hand, the RLS adaptive filter showed similar results to those of the ground truth in all bands. Therefore, we recommend the use of an RLS adaptive filter when analyzing the characteristics of EEG according to random vibration using RP.

We also compared the performance for 4 different cases in the presence of actual movement artifacts. As shown in Figures 9, 10, the coherence without employing the adaptive filter was significantly higher than that with the adaptive filter. Note also that, comparing Figures 10A,C, ICA with ASR did not play a role in removing movement artifact in this study. This is because the ICA decomposition alone is not capable of detecting movement artifacts when random vibrations causing movement artifacts generate spikes, as shown in Figure 8A. The ICA is generally less effective for transient and nonbiological artifacts (Chang et al., 2019). However, in gait analysis there are several studies that have used ICA after ASR to remove movement artifacts (Bulea et al., 2015; Oh et al., 2021). To remove movement artifacts, they used a variance threshold of 3 standard deviation, which is stricter than the case of the variance threshold of ASR in this study. However, Chang et al. (2019) demonstrated that a variance threshold below 7 standard deviations may remove too much brain activities. For these reasons, we can conclude that the removal of the movement artifacts using the RLS adaptive filter is better to minimize the loss of the EEG signal, and the use of adaptive filtering was successful in the case of WBV.

### 4.2 Mental stress analysis under random vibration

We assessed mental stress using the changes in FAA and relative power indices under static and random vibration conditions. First, the FAA decreases and becomes negative as the magnitude of the random vibration increases, as shown in Figure 12A. According to the literature (Giannakakis et al., 2022), a decrease in FAA is the most consistent stress indicator in stress studies. This is because the left anterior region of the brain is associated with approach-type emotions (positive emotion), and the right anterior region is associated with avoidance-type emotions (negative emotion). Therefore, the right alpha activity is generally greater than the left alpha activity during stress. Therefore, based on the decrease in FAA, it is reasonable to conclude that random vibration induces mental stress. We also conclude that the greater the magnitude of the random vibration, the more severe the mental stress.

Among the relative power indices, the alpha and beta band power in the frontal region are known to be the major indicators when an individual is under stress (Giannakakis et al., 2022). The alpha band is prominent in a calm and relaxed state and is characterized by a decrease under stressed conditions. Our results are consistent with the existing literature and also show the largest changes when an individual is subjected to vibration compared with other frequency bands, as shown in Figure 12D. The beta band is generally activated during a state of high alertness or attention and tends to increase during stress. However, our study did not reveal any statistically significant differences between the static and vibration conditions, as shown in Figure 12E. Furthermore, the mean of beta band power decreases slightly under vibration. In this study, the screen remained stationary in the vibration conditions while the motion simulator moved up and down. As visual attention is reduced during vibration, it is natural that the visual attention can lead to a slight decrease in beta band power. For the delta and theta bands, no significant difference was observed between the static and vibration conditions. For the gamma band, there is a study showing that gamma band power increases in stressful situations (Minguillon et al., 2016), and our study also showed that gamma band power increased with increasing vibration magnitude. However, it should also be noted that the gamma band is not as crucial an indicator of stress as the alpha or beta bands.

When a random vibration was applied to the human body, the FAA and alpha band power, which are the major known indicators of a stress situation, showed a decreasing tendency. The greater the magnitude of the vibration, the more these two indicators decrease, indicating that the stress is more severe. On the other hand, beta band power, another known key indicator, showed no statistically significant difference between the static and vibration conditions. Finally, a slight increase in gamma band power was observed in this study as the magnitude of vibration increased. In a future study, we will analyze the mental stress for different frequencies of sinusoidal vibration using EEG, similar to the frequency-dependent discomfort analysis in the vibration field. We also plan to measure and quantify stress in different aspects using EEG and other physiological signals [e.g., electrocardiogram (ECG), electromyography (EMG), and galvanic skin response (GSR)]. Finally, we will analyze the correlation between self-reports and physiological signals.

While the present study offers a novel contribution and notable advantages, certain limitations should be acknowledged. Our investigation was confined to stress induced by random vibration, without considering potential diversity factors such as gender, health status, vibration sensitivity, or long-term effects. Nonetheless, the findings may be applicable to scenarios involving passengers engaged in non-driving related activities (NDRA) and/or urban air mobility (UAM) operations.

## 5 Conclusion

In this study, we demonstrated the necessity of the adaptive filter by comparing the effect of the artifact in the EEG signal influenced by head movement, and successfully removed the artifacts. Using the EEG signals, we analyzed 22 occupants subjected to whole-body vibration in spectral and spatial aspects. As a result, we found that the activities detected in the frontal electrodes showed significant differences between the static and vibration conditions. In addition, we assessed mental stress for 3 different levels of the random vibration using EEG, and found that frontal alpha asymmetry (FAA) and alpha band power, which are key indicators of stress, decreased when exposed to vibration. Based on the literature, which states that stress increases with a decrease in FAA and alpha band power, it can therefore be concluded that random vibration causes mental stress, and that the stress increases with the magnitude of the vibration. The analysis of stress using EEG under random vibration is expected to improve the reliability of ride comfort assessment by complementing the shortcomings of the acceleration method and the survey method. For example, the acceleration method cannot assess stress from other stressors in the vehicle, such as noise, ventilation, scent, and temperature changes, and the survey method is not suitable for real-time assessment. However, EEG provides excellent temporal resolution, allowing real-time monitoring and the assessment of stress changes caused by various factors. Therefore, the stress assessment using EEG can provide a more comprehensive and reliable assessment of ride comfort alongside acceleration and survey methods. In addition, while the literature on vehicle stress using physiological signals has mostly focused on the driver, the current study analyzed the stress experienced by non-driving passengers. From a practical point of view, this research has implications for the development of autonomous driving technology, reducing the need to drive and increasing the importance of in-vehicle infotainment.

## Data availability statement

The raw data supporting the conclusions of this article will be made available by the authors, without undue reservation.

## Ethics statement

The studies involving humans were approved by Kyungpook National University Institutional Review Board (2023-0041). The studies were conducted in accordance with the local legislation and institutional requirements. The participants provided their written informed consent to participate in this study.

## Author contributions

B-GS: Conceptualization, Formal analysis, Investigation, Methodology, Resources, Software, Validation, Visualization, Writing – original draft, Writing – review & editing. NK: Conceptualization, Funding acquisition, Project administration, Supervision, Validation, Writing – review & editing.
